# Attachment and Relational Dynamics in Residential Care Contexts: Perceptions of Reference Adults and Implications for Resilience

**DOI:** 10.3390/bs16091666

**Published:** 2026-09-16

**Authors:** Dora Pereira, Jessica Abreu, Pedro Costa, Margarida Pocinho

**Affiliations:** 1 University Research Center in Psychology, Department of Psychology, University of Madeira, 9020-105 Funchal, Portugal; dora.pereira@staff.uma.pt; 2 Department of Psychology, University of Madeira, 9020-105 Funchal, Portugal; 2107420@student.uma.pt (J.A.); 2149424@student.uma.pt (P.C.)

**Keywords:** attachment, relational dynamics, adolescents, reference adults, resilience, trauma, residential care

## Abstract

Residential care settings provide not only protection but also therapeutic opportunities for children and adolescents exposed to relational adversity. This study examined attachment-related behaviors, relational dynamics, and perceptions of reference adults in residential care institutions in Portugal. Participants included 33 adolescents aged 12–18 years and 20 adults working in residential care institutions. Adolescents completed the Inventory of Attachment in Childhood and Adolescence, while professionals completed the Observation Checklist and provided qualitative descriptions of adolescents’ functioning and intervention strategies. The results revealed a predominance of non-secure attachment profiles, particularly ambivalent attachment, alongside considerable heterogeneity in observed attachment-related behaviors. Avoidant attachment emerged as a significant predictor of increased observational mental health risk, whereas observed secure attachment-related behaviors were negatively associated with psychological vulnerability. Qualitative findings highlighted the importance of emotionally responsive, consistent, and trusting relationships with reference adults, as well as the value of emotional validation, clear boundaries, and attachment-informed caregiving practices. The high heterogeneity of self-reported attachment representations and observational measures underscores the complexity of attachment functioning in trauma-exposed populations and supports the use of multi-method assessment approaches. Overall, the qualitative findings suggest that resilience in residential care is fundamentally relational and may be strengthened through stable, emotionally attuned relationships with caregivers. This study reinforces the importance of attachment-informed and trauma-informed practices in promoting psychological well-being, emotional regulation, and positive developmental trajectories among adolescents living in residential care.

## 1. Introduction

Residential care remains a fundamental protective measure within contemporary child welfare systems, particularly when children and adolescents cannot safely remain in their biological families. Although family-based alternatives are increasingly promoted internationally, residential care continues to be one of the most frequently implemented protective responses in several European countries, including Portugal (European Commission, 2024; Instituto da Segurança Social, 2024). Placement in residential care aims to protect children from situations of abuse, neglect, domestic violence, parental incapacity, and other forms of significant risk. However, such interventions often require separation from primary caregivers and adaptation to unfamiliar environments, creating a paradox in which a measure designed to ensure safety may initially generate further experiences of insecurity (Schofield et al., 2017).

Attachment theory provides a valuable framework for understanding this paradox. According to Bowlby (1988), children develop internal working models of themselves, others, and relationships through repeated interactions with attachment figures. These models guide expectations regarding care, protection, trust, and emotional regulation throughout development. When caregivers respond consistently and sensitively to children’s emotional needs, secure attachment representations are more likely to emerge, fostering resilience, socioemotional competence, self-regulation, and psychological well-being (Ainsworth, 1991; Cassidy & Shaver, 2016). Conversely, experiences of abuse, neglect, rejection, inconsistency, or frightening caregiving environments contribute to the development of insecure or disorganized attachment patterns, often characterized by difficulties in affect regulation, interpersonal trust, behavioral adaptation, and mental health functioning (Figueiredo, 2023; Lyons-Ruth & Jacobvitz, 2016).

Children entering residential care have frequently experienced significant relational adversity. In Portugal, the most common reasons for residential placement include neglect, exposure to domestic violence, emotional maltreatment, inadequate parental supervision, and other circumstances compromising children’s safety and development (Instituto da Segurança Social, 2024). These experiences are particularly harmful because they occur within relationships that should provide protection, emotional security, and support. As a result, children and adolescents in residential care often present insecure or disorganized attachment representations at substantially higher rates than those observed in community populations (Schuengel et al., 2014; Steinhauer, 1991). Such attachment difficulties have been consistently associated with increased vulnerability to depression, anxiety disorders, post-traumatic stress symptoms, behavioral dysregulation, substance misuse, peer relationship difficulties, and attachment-related psychopathology (Fearon et al., 2010; Lyons-Ruth & Jacobvitz, 2016).

The developmental consequences of chronic relational adversity have increasingly been conceptualized within trauma-informed frameworks. Van der Kolk (2014) proposed the construct of Developmental Trauma Disorder to describe the complex psychological, emotional, behavioral, and relational difficulties frequently observed in children exposed to prolonged experiences of abuse, neglect, and disrupted caregiving relationships. Research consistently demonstrates that relational trauma affects not only emotional regulation but also cognitive functioning, interpersonal relationships, self-concept, and adaptive developmental trajectories (Cook et al., 2005; Van der Kolk, 2014).

Within this context, residential care should not be viewed solely as a protective intervention but also as a potentially therapeutic environment capable of promoting recovery from relational trauma and fostering resilience processes.

Although resilience has been acknowledged as a relevant process in the field of child protection, it is only in the last two decades that researchers have focused their efforts on studying this issue in residential care, particularly the concept of resilience, the factors that foster resilience in residential care, and the mechanisms through which resilience is promoted. In their review, Lou et al. (2018) considered that the literature lacks a clear definition of resilience in residential care and that evidence of resilience has been acknowledged through the absence of or decrease in problematic behaviors (e.g., delinquent acts) but also through the increase in positive features or processes (e.g., problem solving or self-regulation skills). The same review notes that more recent studies conceive resilience ecologically as a dynamic process determined by factors from different ecological levels or systems. More specifically, the authors identified, at an individual level, age, gender and frequency of abusive or neglect experiences; at the family level, acceptance, rejection or control from parental figures; and at a mesosystemic level, school engagement or the time spent in a residential care setting. From the perspective of adolescents in residential care, the same three levels of factors, individual, out of care (e.g., contact with family) and residential care (e.g., quality of staff support), were also identified as the most important in fostering resilience (Pinheiro et al., 2024) in that specific setting. However, adolescents may perceive their resilience assets as more negative (compared with adolescents from a community sample), specifically in self-efficacy, empathy, and goals and aspirations (Lemos et al., 2021). From the perspective of professional staff working in residential care homes, the main factors associated with adolescents’ resilience in residential care are contact with their families and their individual characteristics. Still, they also highlight the importance of the support and empathy provided by staff carers in the homes (Pinheiro et al., 2026). Good resilience processes were reported as associated with different therapeutic and supportive interventions, such as the construction of positive narratives about their own story, nurturing self-compassion, self-value, and self-belief, and establishing trust and affective relations with staff in homes (Parry et al., 2023). Mishra (2024) suggests, as best practices to promote resilience, that all significant figures for children in care should make efforts to ensure social recognition, opportunities to develop relevant skills for life after care, and cohesion and prosocial behaviors during their stay in care.

Effectively, contemporary attachment-informed approaches emphasize that stable, predictable, and emotionally responsive relationships can provide corrective emotional experiences that contribute to the reorganization of insecure internal working models and the development of greater emotional security (Golding et al., 2016; Schuengel et al., 2014). Consequently, residential care professionals may become significant secondary attachment figures, particularly when they engage in consistent, nurturing, and attuned caregiving practices.

Accumulating evidence suggests that the quality of adult–child relationships within residential care settings is one of the strongest predictors of positive developmental outcomes. Children and adolescents who perceive caregivers as available, supportive, and emotionally responsive tend to demonstrate greater resilience, improved emotional regulation, better behavioral adjustment, and enhanced psychological well-being (Graham, 2005; Lou et al., 2018; Pinheiro et al., 2026). Similarly, a recent systematic review about attachment bonds in residential care (Martínez-Usarralde et al., 2025) indicated that although children in residential care generally present elevated levels of emotional and behavioral difficulties, sensitive and relationally available caregivers can significantly improve socioemotional adaptation and long-term developmental outcomes.

The effectiveness of attachment-informed residential care depends largely on professionals’ ability to recognize and accurately interpret attachment-related behaviors. Many behaviors commonly observed in residential care settings—including withdrawal, avoidance of closeness, excessive dependency, oppositionality, emotional dysregulation, or apparent indifference—can be understood as adaptive relational strategies developed in response to previous experiences of adversity (Golding et al., 2015, 2016). Without an attachment-informed perspective, professionals may focus primarily on behavior management while overlooking the relational and emotional needs underlying these behavioral manifestations.

Understanding how professionals perceive and respond to attachment-related signals is therefore of particular importance. Adults’ interpretations of children’s behaviors influence daily interactions, caregiving responses, opportunities for emotional co-regulation, and the overall quality of the relational environment experienced by young people in care. The theoretical and empirical literature consistently highlights adult relational sensitivity as a central mechanism through which emotional security, trust, resilience, and adaptive functioning are promoted in non-familial care contexts (Bowlby, 1988; Golding et al., 2016; Schuengel et al., 2014). Reference adults who are capable of recognizing attachment needs and responding sensitively can provide a secure relational base from which children and adolescents gradually develop greater emotional stability and interpersonal competence.

Despite the growing recognition of attachment-informed and trauma-informed practices within child welfare systems, empirical research examining how residential care professionals perceive attachment-related behaviors in everyday interactions remains limited. Morison et al. (2020) acknowledge the gap between theory and practice, noting that participants in their study look forward to “naturally” (p. 9) establishing positive relationships with children in care, “with theory in background” (p. 9), but cannot coherently link their daily practices to attachment theory. This gap is particularly relevant because professionals’ perceptions directly influence intervention strategies and the quality of relationships established with children and adolescents. Observational tools based on behavioral indicators that professionals can observe in their daily practices, strongly supported in attachment theory, can inform and guide professionals’ interpretations about adolescents’ behavior and relational practices. Vorria et al. (2015) asserted that observational measures should be used to overcome the limitations of self-report measures as scales or interviews in residential care. However, the scoping review of Shields (2025) focused specifically on attachment development and attachment difficulties in out-of-home care settings and does not include any article with observational measures directed toward adolescents. The search for recent articles (in the last 5 years) that include observational measures of adolescents’ attachment-related behaviors in residential care settings did not return any results.

To address this gap, the present exploratory study examines attachment-related behaviors and relational dynamics among adolescents living in residential care institutions in Madeira, Portugal, based on a double-informant perspective. Specifically, this study integrates adolescents’ self-reported attachment representations with observational data provided by educators and other reference adults, aiming to explore how attachment needs are perceived and responded to within everyday residential care interactions. By combining standardized assessment measures with professionals’ perspectives, this research seeks to contribute to a deeper understanding of attachment-informed practice and its role in promoting resilience, socioemotional adjustment, and psychological well-being among adolescents exposed to relational adversity. A multi-method approach was adopted because attachment-related functioning in residential care involves complementary internal, behavioral, and relational–contextual dimensions that cannot be fully captured through a single source of information. The IVIA was used to assess adolescents’ self-reported attachment representations, providing access to their subjective perceptions of relational security and insecurity. The Observation Checklist complemented this perspective by capturing attachment-related behaviors and socioemotional functioning as observed by professionals during everyday interactions. The use of both sources of information, adolescents and carers, is coherent with the relational nature of attachment processes, as to consider only one subject’s perspective would not be sufficient to understand a relational dynamic. Finally, the qualitative narratives provided contextual information regarding professionals’ perceptions of adolescents’ relational and emotional functioning and the caregiving strategies considered effective in responding to their needs. Thus, rather than representing redundant assessments, the three sources of information were used to examine complementary aspects of attachment-related functioning and to provide a more comprehensive understanding of relational dynamics within residential care.

### Objectives

The present exploratory study had four main objectives:

1. To assess attachment representations among adolescents living in residential care.
2. To examine attachment-related behaviors observed by educators and other reference adults within residential care settings.
3. To examine the associations between attachment patterns, relational functioning, and indicators of psychological vulnerability.
4. To analyze professionals’ perceptions of effective caregiving and supportive practices and identify attachment-informed processes that may contribute to resilience, socioemotional stability, and psychological well-being among adolescents exposed to relational trauma.

## 2. Materials and Methods

### 2.1. Study Design

This study employed an exploratory, cross-sectional, multi-method design integrating quantitative and qualitative sources of information to examine attachment-related functioning and relational dynamics among adolescents living in residential care. The quantitative component comprised adolescents’ self-reported attachment representations assessed with the Inventory of Attachment in Childhood and Adolescence (IVIA), sociodemographic information, and professionals’ observations of attachment-related behaviors and socioemotional functioning using the Observation Checklist.

Exploratory Mann–Whitney U tests were used to examine whether adolescents classified as having a predominantly secure versus non-secure self-reported IVIA profile differed in secure, ambivalent, and avoidant attachment-related behaviors independently rated by professionals using the Observation Checklist. Because group membership was derived from the IVIA whereas the compared variables were obtained from the Observation Checklist, these analyses did not compare participants on the same scores used to establish group membership. Given the small sample, these comparisons were interpreted as exploratory.

The qualitative component consisted of narrative records provided by professionals regarding adolescents’ psychosocial and relational functioning and caregiving strategies perceived as effective.

The different sources of information were analyzed using methods appropriate to their nature. Quantitative data were examined through descriptive, correlational, group comparison, and regression analyses, whereas the professional narratives were examined using inductive qualitative content analysis. The quantitative and qualitative components were subsequently considered together at the interpretative level to provide complementary perspectives on adolescents’ internal attachment representations, observable relational behaviors, and the relational context of residential care. This design was intended to provide a more comprehensive account of attachment-related functioning than would be possible using a single method or informant.

### 2.2. Participants

Convenience sampling was employed and the sample included 33 adolescents living in five residential care institutions in Madeira, Portugal (Table 1). The adolescents were observed by educators and care professionals working directly with them in daily care contexts. Age data were available for 33 participants, ranging from 12 to 18 years old, with a concentration in mid-adolescence (M = 15 years old), comprising 18 females and 15 males. The 20 reference adults were mostly female (n = 15; 75%), with a mean age of 40 years (DP = 8), a minimum of 27 years, and a maximum of 59 years, and with bachelor’s and/or master’s degrees. Each adolescent was rated by one reference professional rather than by a staff team. Professionals were selected on a voluntary basis and received training in the use of the Observation Checklist before completing the assessment. Each professional rated an adolescent with whom they were familiar through routine residential care interactions. The 33 adolescents were distributed across the five participating residential care institutions as follows: Institution A, n = 10; Institution B, n = 10; Institution C, n = 8; Institution D, n = 4; and Institution E, n = 1. The distribution of ratings across professionals was as follows: Institution A, n = 8; Institution B, n = 8; Institution C, n = 1; Institution D, n = 2; and Institution E, n = 1. Across the full sample, thirteen professionals rated one adolescent each, six professionals rated two adolescents each, and one professional rated eight adolescents.

The length of stay in residential care ranged from 1 month to 8 years (M = 24 months), with most adolescents having been placed for less than two years, although a small number had experienced long-term placements exceeding four years. The most represented school years were 8th grade (21.2%) and 10th grade (15.2%); 9 participants (27.3%) did not write their school grade. Most participants did not present identified special educational needs (87.9%). However, a small proportion of participants had documented needs, including Attention-Deficit Hyperactivity Disorder (ADHD) (6.1%), intellectual functioning difficulties (3.0%), and one case receiving specialized educational support. Information about the adolescents was primarily reported by educators (60.0%), followed by psychologists (20.0%), direct care assistants (15.0%), and a social worker (5.0%).

### 2.3. Measures

#### 2.3.1. Sociodemographic Questionnaire

A sociodemographic questionnaire was specifically developed for this study to collect information on participants’ age, gender, educational level, grade retention history, and adverse childhood events. Academic achievement was operationalized using teacher-reported academic performance indicators (e.g., grades, retention) and complemented by child self-reports in an age-appropriate format. Although indirect, this approach provided functional quantitative indicators of school performance.

#### 2.3.2. Inventory of Attachment in Childhood and Adolescence (IVIA)

Attachment representations were assessed using the Inventory of Attachment in Childhood and Adolescence (IVIA), developed by Carvalho et al. (2006).

The IVIA is grounded in Bowlby’s attachment theory and assesses three attachment dimensions: secure attachment, insecure ambivalent attachment, and insecure avoidant attachment. The final validated version comprises 24 items distributed equally across the three dimensions. Responses are rated on a five-point Likert scale (1 = Never to 5 = Always), with higher scores indicating stronger endorsement of behaviors and perceptions associated with each attachment style. The IVIA was validated in a Portuguese non-clinical sample of 577 participants aged 7–17 years. Exploratory factor analyses supported a three-factor structure consistent with the theoretical model, and internal consistency indices were reported as adequate. In the present study, the self-report version was administered to children/adolescents. The scoring of the Inventory of Attachment in Childhood and Adolescence (IVIA) followed the structure established in the validated Portuguese version of the instrument (Carvalho et al., 2006). The questionnaire comprises 24 items organized into three attachment dimensions: secure attachment, ambivalent attachment, and avoidant attachment. Each dimension is represented by eight items. Secure attachment was calculated as the mean of items 1, 4, 7, 10, 13, 16, 19, and 22. Ambivalent attachment was calculated as the mean of items 2, 5, 8, 11, 14, 17, 20, and 23. Avoidant attachment was calculated as the mean of items 3, 6, 9, 12, 15, 18, 21, and 24. All items were rated on a five-point Likert scale ranging from 1 (Never) to 5 (Always), with higher scores indicating stronger endorsement of behaviors and relational perceptions associated with each attachment pattern. For each participant, mean scores were computed for the three attachment dimensions, allowing for a comparison of attachment profiles within the sample.

For descriptive purposes, in the present study, a predominant attachment profile was identified according to the highest mean score across the three IVIA dimensions. When equivalent highest scores occurred across dimensions, no predominant profile was assigned. This categorical classification was used descriptively and should not be interpreted as a validated diagnostic classification derived from the IVIA.

The interpretation of IVIA scores follows the response scale of the instrument, which ranges from 1 (Never) to 5 (Always). Mean scores were calculated for each attachment dimension, allowing for an interpretation of the intensity of each attachment pattern. Values between 4 and 5 indicate a high level of endorsement of the behaviors and perceptions associated with the respective attachment dimension, whereas scores around 3 reflect moderate levels. Mean scores between 1 and 2 indicate low endorsement of the corresponding attachment characteristics. For example, a mean score of 3.6 on the secure attachment dimension reflects a moderate level of perceived relational security, whereas a mean score of 3.9 on the ambivalent dimension indicates relatively elevated relational anxiety and sensitivity to interpersonal dynamics. A mean score of 2.5 on the avoidant attachment dimension suggests moderate tendencies toward emotional distancing or reduced help-seeking in relational contexts.

This scale reveals good reliability in this study, with Cronbach’s alpha from 0.71 in the secure dimension, and 0.91 in the ambivalent dimension and 0.91 in the avoidant dimension.

#### 2.3.3. Observation Checklist

Attachment-related behaviors and socioemotional functioning were assessed using the Observation Checklist developed by Golding et al. (2015). This structured instrument supports systematic observation of adolescents aged 11–16 years in educational settings, particularly focusing on behaviors associated with insecure or disorganized attachment patterns. In the present sample, nine participants were older than this age range (eight aged 17 and one aged 18). The checklist was used as a structured observational and reflective tool rather than as a diagnostic instrument. Because its validity for adolescents aged 17–18 has not been established, analyses involving these participants should be interpreted cautiously. A sensitivity analysis excluding participants older than 16 years was therefore conducted to examine whether their inclusion materially affected the main regression findings.

The checklist includes six sections: general behavior, observational mental health risk indicators, classroom-related behaviors, social relationship with peers, attachment behaviors, and emotional state. Each section includes several pairs of opposite behaviors (e.g., resist limits, do not comply/easily compliant, accept limits with low protest) and observers should classify each one considering if the adolescent is near one of the extremes or in the middle. The first item of each pair describes behaviors more common in adolescents with insecure ambivalent internal working models, and the second item describes behaviors associated with insecure avoidant internal working models of attachment (iwma). The middle is related to behavior common in the majority of children of the same age—the secure internal working models. The dominance of one of the extremes or the middle suggests a dominant category of behaviors representing a type of iwma. The non-dominance suggests a disorganized iwma as the dominant type of behavior. This checklist is not a diagnostic instrument but rather a reflective monitoring tool intended to guide educational responses. In the present study, it was completed by educators who were familiar with the child and had observed them over an extended period (two months). At the end of each section, the professional was asked to provide a descriptive narrative of the behavioral specificities that justify the classification attributed. These qualitative data were analyzed as described in the Procedures Section.

For each adolescent, the attachment-related observational dimensions and the observational mental health risk indicator were derived from the same Observation Checklist completed by the same reference professional. Thus, these measures represent different domains within a common professional-report method rather than independent informant assessments.

Observational mental health risk indicators. An exploratory observational risk indicator was derived from the Observation Checklist to summarize the frequency of behaviors potentially indicative of psychological or behavioral vulnerability. The indicator included checklist items referring to risk-related behaviors, such as substance use, self-harm, and addictive or other potentially harmful behaviors. For each adolescent, the frequencies recorded across these items were combined to obtain an overall observational risk score, with higher scores indicating a greater frequency of observed risk-related behaviors. This score was not intended to represent a diagnostic measure or a validated psychometric scale, but rather a descriptive composite indicator of behavioral and psychological vulnerability based on professionals’ observations. Because the included items represent heterogeneous risk behaviors rather than interchangeable manifestations of a single latent construct, internal consistency was not considered an appropriate reliability criterion for this composite indicator. For the exploratory observational mental health risk indicator, challenging/risk behavior items from the Observation Checklist (five point Likert) were considered (Golding et al., 2015): sexualized behaviors—language, gestures or body language; substance misuse—drugs, alcohol or other substances; verbally challenging behaviors—attitude/tone of voice; threating behavior—verbally/physically; threating behavior—weapon use; absconding/self exclusion; educational refusal; risk of use ICT—social media, games, etc. For each item, the response categories A–E were coded from 4 to 0, respectively: A (almost always) = 4, B (sometimes) = 3, C (as student at same stage of development) = 2, D (hardly ever) = 1, and E (never) = 0. The indicator was calculated as the mean of the coded item scores: (A × 4 + B × 3 + C × 2 + D × 1 + E × 0)/5, with higher values indicating greater observed mental health-related risk. This composite was used as an exploratory observational indicator and should not be interpreted as a diagnostic measure or validated psychometric scale. Given that the items represent heterogeneous risk behaviors, internal consistency was not considered an appropriate reliability criterion.

### 2.4. Procedures

This study followed all ethical and institutional requirements for research involving minors. Approval was first obtained from the University of Madeira. Subsequently, ethical approval was requested from the relevant ethics committee to ensure compliance with established principles of human research ethics, including informed consent, confidentiality, voluntary participation, and the minimization of potential risks. After ethical clearance, institutional authorization was obtained from residential care institutions. Written informed consent was obtained from parents, legal guardians, or institutional caregivers prior to participation.

Data collection was conducted in residential settings in collaboration with institutional staff. Adolescents completed the IVIA and the adapted sociodemographic questionnaire in supervised sessions. Educators independently completed the Observation Checklist and academic performance indicators after training for 3 h focused on the observational procedures; educators were instructed to register the more usual behavior of the adolescents (in order to identify behavioral patterns and not casual events) and also events that can impact the attachment behavioral system (such as changes in contact with family, disease periods, etc.).

### 2.5. Statistical and Qualitative Data Analysis

Statistical analysis. All data were anonymized prior to statistical analysis.

Quantitative analyses were conducted using IBM SPSS Statistics, version 29.0. Descriptive statistics were used to characterize the sample and the main study variables. Categorical variables were summarized using frequencies and percentages, n (%), whereas continuous variables were described using means and standard deviations, M (SD), together with minimum and maximum values where informative. Statistical significance was set at *p* < 0.05, and all statistical tests were two-tailed. Effect sizes and 95% confidence intervals (CIs) were reported for the main inferential analyses to complement significance testing and provide information regarding the magnitude and precision of the observed effects. For Mann–Whitney U comparisons, effect size was expressed as *r*. For the multiple linear regression, unstandardized regression coefficients (*B*) are presented with their corresponding 95% CIs, together with standardized coefficients (β), and Cohen effect size estimate.

Spearman’s rank-order correlations (ρ) were used to examine associations between observational mental health risk indicators, observed attachment-related behaviors, self-reported attachment dimensions, and time in residential care.

Given the small sample size, the regression analysis was intentionally restricted to a parsimonious set of attachment-related predictors and sex. The predictors were not specified in a preregistered analysis plan; therefore, the regression should be regarded as exploratory rather than confirmatory. Avoidant and secure IVIA dimensions were included to represent adolescents’ self-reported insecure and secure attachment-related representations, respectively, whereas the secure checklist dimension provided an independent observational indicator of secure relational behavior. Sex was retained as a basic demographic covariate. To reduce overparameterization, additional attachment dimensions and sociodemographic variables were not entered into the model. Accordingly, individual *p* values are interpreted cautiously, with emphasis placed on the direction and magnitude of associations and the exploratory nature of the model.

A multiple linear regression analysis was conducted to examine predictors of observational mental health risk indicators. Predictors were entered simultaneously using the Enter method in SPSS version 29.0. Given the limited sample size (N = 33) and the exploratory nature of this study, a parsimonious model was specified to reduce the risk of overparameterization. Attachment-related predictors and sex were entered simultaneously, with sex included as a sociodemographic covariate to account for potential sex-related differences in psychological vulnerability. Other sociodemographic variables were not simultaneously included in order to limit model complexity relative to the available sample size. Regression assumptions were examined prior to interpretation of the model. Approximate normality of residuals and homoscedasticity were evaluated through inspection of residual diagnostics, multicollinearity was assessed using variance inflation factors (VIFs), and independence of residuals was examined using the Durbin–Watson statistic.

Given the limited sample size, an a priori sensitivity analysis was considered more informative than a conventional post hoc power analysis. For a multiple regression model with four predictors, N = 33, α = 0.05, and 80% power, the minimum detectable overall effect was approximately Cohen’s f^2^ = 0.43 (corresponding to approximately R^2^ = 0.30). Thus, this study was adequately powered only to detect relatively large overall effects, and smaller effects may have remained undetected. The regression analysis was therefore considered exploratory.

There were no missing data for the quantitative variables included in the statistical analyses; therefore, no imputation or other procedures for handling missing quantitative data were required.

Qualitative analysis. The narrative records provided by professionals were analyzed manually using inductive qualitative content analysis, following the general procedures described by Elo and Kyngäs (2008). This approach was selected because the qualitative material consisted of brief and heterogeneous open-ended narrative records and the aim was to identify and organize recurrent content concerning adolescents’ psychosocial and relational functioning and caregiving strategies perceived as effective. The analysis proceeded through three broad phases: preparation, organization, and reporting. First, the available narratives were read repeatedly to obtain an overall understanding of the material. Second, relevant meaning units were identified and coded according to their manifest content. Codes expressing similar meanings were compared and inductively grouped into broader categories, which were subsequently reviewed and refined to represent recurrent patterns across the narratives. The coding and categorization process was conducted manually by two researchers. Initial coding was performed by the last author and subsequently reviewed by the first author. The researchers compared and discussed the codes and emerging categories, and differences in interpretation were resolved through discussion and consensus. No qualitative data analysis software was used. Finally, the resulting categories were described and interpreted in relation to the study objectives. Blank narrative fields were treated as missing qualitative data rather than as evidence of the absence of a behavior, experience, or category. Given the brevity and heterogeneity of the narratives and the substantial number of blank responses, the qualitative findings were interpreted as exploratory.

## 3. Results

### 3.1. Relational Environment in Residential Care

#### 3.1.1. Previous Residential Care Placements

Information about previous placements in residential care institutions was available for 33 adolescents. The majority of participants (75.8%) reported no prior placement in other residential care facilities, whereas 24.2% indicated that they had previously been placed in another institution.

Among those with previous placements (n = 8), most adolescents (87.5%) had experienced one previous placement, while 12.5% reported two previous placements.

These results suggest that the majority of adolescents in the sample were experiencing their first residential care placement, although a smaller proportion had already undergone placement transitions within the care system.

#### 3.1.2. The Presence of a Reference Figure

Most adolescents were reported to have a reference figure within the residential care setting. Specifically, 90.9% of participants were described as having an identified reference figure, while 9.1% did not.

When professionals specified who fulfilled this role (n = 30), the most frequently mentioned figures were a reference educator (38.1%), a reference technical staff member (19.0%), educators (4.8%), and doctors or other professionals (4.8%).

Some responses indicated shared reference figures, such as an educator together with a psychologist or other technical staff.

Overall, these findings highlight the central role of educators and technical staff as key relational figures for adolescents living in residential care settings.

#### 3.1.3. Perceived Support from Professionals

Most adolescents reported feeling heard by professionals in the residential care setting. Specifically, 60.6% indicated that they always feel listened to, while 36.4% reported feeling listened to only sometimes, and 3.0% stated that they rarely feel heard.

Regarding trust in professionals, 54.5% of adolescents reported that they trust the professionals in the residential care institution, whereas 42.4% indicated that their trust depends on the situation, and 3.0% reported not trusting them.

These findings suggest that relationships with professionals are generally perceived positively, although trust may vary depending on circumstances.

#### 3.1.4. Relationships with Other Adolescents

The majority of participants reported positive relationships with other adolescents living in the institution. More than half described these relationships as good (51.5%), while 18.2% reported very good relationships, and 30.3% characterized them as regular.

Overall, these results indicate that peer relationships within residential care settings are generally perceived as satisfactory.

#### 3.1.5. Contact with Family

Family contact remained relatively frequent for many adolescents. Among respondents, 60.6% reported communicating with family members daily, 27.3% weekly, 6.1% occasionally, and 6.1% reported no contact.

These findings indicate that family relationships often remain active despite residential placement.

#### 3.1.6. Seeking Support When Feeling Sad

When experiencing sadness, 75.8% of adolescents reported talking to someone within the residential care setting, whereas 24.2% reported not doing so.

Among those who seek support, the main sources of support were technical staff members (44.0%), educational staff (32.0%), and other adolescents living in the institution (24.0%).

This suggests that professionals in residential care institutions play a key role as emotional support figures.

#### 3.1.7. Support Outside the Institution

Most adolescents (90.9%) reported having someone outside the institution they could rely on, while 9.1% reported not having external support. Among those who reported external support, the most frequently mentioned sources were parents (36.7%), siblings or other relatives (33.3%), close friends (13.3%), and other supportive figures, including former caregivers or professionals (16.7%).

These results (see Table 2) highlight the importance of maintaining social networks beyond the residential care environment.

### 3.2. Emotional Regulation

Emotional regulation was reported as an area of difficulty for many adolescents. While 30.3% (n = 10) were described as able to regulate their emotions, the majority (51.5%; n = 17) were described as regulating their emotions only sometimes, and 18.2% (n = 6) were described as having difficulties controlling emotional responses. These findings suggest that difficulties in emotional regulation constitute a significant developmental challenge for a substantial proportion of adolescents living in residential care.

Participation in emotional development programs within the residential care institutions was reported by 39.4% (n = 13) of adolescents, while 42.4% (n = 14) stated that they had not participated, and 18.2% (n = 6) were unsure.

This indicates that structured emotional development interventions are present but not universally implemented across participants.

Overall, the findings suggest that adolescents living in residential care generally report positive relationships with professionals and peers, maintain contact with family members, and rely on multiple sources of emotional support. However, emotional regulation difficulties remain common, highlighting the importance of targeted socioemotional development programs within residential care settings.

### 3.3. Attachment Inventory of Childhood and Adolescents Results

Attachment representations were assessed using the Inventory of Attachment in Childhood and Adolescence (IVIA) by adolescent’s self-report. Descriptive statistics for the three attachment dimensions—secure attachment, ambivalent attachment, and avoidant attachment—are presented in Table 3.

The results indicate moderate levels across all attachment dimensions, with slightly higher scores for ambivalent attachment (M = 3.19, SD = 0.61), followed by secure (M = 3.07, SD = 0.73) and avoidant (M = 2.98, SD = 0.63) attachment.

These findings suggest a predominance of ambivalent relational patterns, reflecting heightened sensitivity to interpersonal dynamics, fear of abandonment, and emotional dependency. Although secure attachment scores were within the moderate range, they did not clearly exceed insecure scores, indicating that perceived relational safety may coexist with underlying insecurity.

Avoidant attachment scores were comparatively lower but still within a moderate range, suggesting the presence of defensive strategies such as emotional distancing and reduced help-seeking behaviors in a subset of participants.

Importantly, the coexistence of moderate secure and insecure attachment scores suggests that adolescents in residential care may experience simultaneous needs for proximity and protection alongside defensive relational strategies, highlighting the complexity of their relational functioning. Overall, the attachment profile of this sample reflects heterogeneous and mixed internal working models and is consistent with populations exposed to relational instability and caregiving disruptions.

Using the descriptive predominant-profile procedure adopted in the present study, the most frequently observed attachment profile was ambivalent attachment, identified in 45.5% of participants (n = 15). Secure attachment was observed in 33.3% of adolescents (n = 11), while avoidant attachment characterized 15.2% of the sample (n = 5). Two participants (6.1%) could not be classified into a predominant attachment category due to equivalent scores across dimensions.

Overall, these findings indicate a predominance of insecure attachment patterns within the sample, particularly ambivalent attachment, suggesting heightened relational sensitivity, concerns regarding interpersonal availability, and increased dependence on attachment figures. Nevertheless, approximately one-third of participants demonstrated predominantly secure attachment representations.

### 3.4. Observation Checklist Results

Descriptive statistics for the observational checklist domains are presented in Table 4.

The secure behavioral dimension showed the highest mean score (M = 19.82, SD = 8.54), indicating that secure attachment-related behaviors were the most frequently observed across participants. In contrast, insecure/ambivalent (M = 11.91, SD = 7.10) and insecure/avoidant behaviors (M = 11.58, SD = 6.47) were also present, although with lower mean values. The range of scores suggests substantial variability across participants in all domains, particularly within the secure dimension (Min = 5; Max = 37), reflecting heterogeneity in observed relational functioning. Similarly, both insecure dimensions showed wide score distributions (ambivalent: Min = 3, Max = 26; avoidant: Min = 2, Max = 24), indicating that while some adolescents exhibited low levels of insecure behaviors, others displayed markedly elevated patterns.

Considering IVIA classifications, a statistically significant difference was observed in ambivalent attachment, U = 42.00, Z = −3.37, *p* < 0.001. The not secure group (mean rank = 22.17) scored higher than the secure group (mean rank = 10.80). The effect size was large (r = 0.59, 95% CI [0.32, 0.77]). The results revealed a highly significant difference in secure attachment, U = 0.00, Z = −4.89, *p* < 0.001. The secure group (mean rank = 26.00) scored higher than the not secure group (mean rank = 9.50). The effect size was very large (r = 0.85, 95% CI [0.72, 0.92]). A significant difference was also found for avoidant attachment, U = 47.00, Z = −3.19, *p* = 0.001. The not secure group (mean rank = 21.89) showed higher scores than the secure group (mean rank = 11.13). The effect size was large (r = 0.56, 95% CI [0.27, 0.75]). Overall, the analyses revealed statistically significant differences across all attachment dimensions, with effect sizes ranging from moderate to very large. However, given the small sample size, these effect estimates should be interpreted cautiously. 

Overall, adolescents with predominantly secure versus non-secure self-reported IVIA profiles also differed in attachment-related behaviors independently observed by professionals. These exploratory findings indicate group-level differences in professionally observed attachment-related behaviors according to adolescents’ predominant self-reported IVIA profile. However, these group-level differences should be considered alongside the generally weak dimensional correlations between the two measurement approaches reported below and should not be interpreted as evidence of strong convergence or validation of the IVIA-derived profiles.

Classification of observational attachment profiles revealed a pattern broadly consistent with the self-report data. Although secure attachment-related behaviors were the most frequently observed at the behavioral level, substantial variability was evident across participants. A considerable proportion of adolescents displayed behaviors associated with ambivalent and avoidant attachment strategies, reflecting diverse ways of managing emotional needs and interpersonal relationships.

### 3.5. Correlation and Regression Analysis

Spearman correlation analyses were conducted to examine associations between observational mental health risk indicators, observed attachment-related behaviors, self-reported attachment representations, and time in residential care (Table 5).

Observational mental health risk indicators were moderately positively associated with ambivalent behaviors (ρ = 0.41) and avoidant behaviors (ρ = 0.36), and negatively associated with secure behaviors (ρ = −0.39), indicating that higher psychological vulnerability is linked to increased attachment insecurity and reduced relational security.

Within observational data, strong negative correlations were found between secure and ambivalent behaviors (ρ = −0.71) and between secure and avoidant behaviors (ρ = −0.62), suggesting that these behavioral dimensions function in opposing directions. No significant association was observed between ambivalent and avoidant behaviors (ρ = 0.11).

Self-reported attachment dimensions (IVIA) were all positively correlated, with moderate associations between secure and avoidant (ρ = 0.58), secure and ambivalent (ρ = 0.52), and avoidant and ambivalent dimensions (ρ = 0.63), reflecting the coexistence of multiple internal attachment dimensions that are usually associated with disorganized attachment representations.

All IVIA dimensions were positively correlated, suggesting the coexistence of multiple internal working models, which may reflect relational ambiguity commonly observed in trauma-exposed populations.

Associations between observational and self-reported measures were generally weak, indicating limited convergence between internal representations and observed behaviors.

Time in residential care showed weak associations across variables, with slight negative correlations with IVIA secure (ρ = −0.22) and ambivalent attachment (ρ = −0.21), suggesting minimal impact of placement duration on internal attachment representations.

Given the limited sample size, an exploratory parsimonious multiple linear regression analysis was conducted to examine whether selected attachment-related indicators and sex were associated with observational mental health risk.

The distribution of the observational mental health risk indicator was also examined. Only one participant had a score of zero, and the distribution showed only slight positive skewness (skewness = 0.32); the Shapiro–Wilk test did not indicate a significant departure from normality (*p* = 0.131).

Influence diagnostics identified one observation with a Cook’s distance above the conventional 4/N threshold (D = 0.189; 4/N = 0.121), although no observation exceeded the leverage threshold of 2(k + 1)/N. A sensitivity analysis excluding this observation produced the same substantive pattern of results (R^2^ = 0.499): avoidant IVIA remained positively associated with mental health risk (B = 1.39, *p* = 0.004), secure checklist remained negatively associated with risk (B = −0.104, *p* < 0.001), and sex remained significant (B = 0.91, *p* = 0.042). Thus, the main findings were not attributable to a single influential observation.

As a further sensitivity analysis, the regression was re-estimated after excluding each of the five institutions in turn. The direction of all regression coefficients remained unchanged across the five reduced-sample models. Model R^2^ values ranged from 0.328 to 0.530. However, the magnitude and statistical significance of individual coefficients varied, particularly when either of the two largest institutions was excluded (N = 23). Secure checklist remained negatively associated with the outcome across all models, whereas avoidant IVIA remained positive but was not statistically significant in all reduced samples. These findings suggest that the directional pattern was not attributable to a single institution, while also indicating limited precision and some sensitivity of individual coefficients to sample composition.

Given the limited sample size (N = 33) and the exploratory nature of this study, a parsimonious model was specified to avoid including an excessive number of predictors relative to the available sample. The attachment-related predictors and sex were entered simultaneously into the model. Sex was included as a sociodemographic covariate to account for potential sex-related differences in psychological vulnerability. Other sociodemographic variables collected through the questionnaire were not simultaneously entered into the model in order to limit model complexity and reduce the risk of overfitting. Accordingly, the regression should be interpreted as an exploratory model rather than as a fully adjusted model controlling for all available sociodemographic characteristics.

The model was significant, *F*(4, 28) = 5.15, *p* = 0.003, explaining 42.4% of the variance (*R*^2^ = 0.42) (Table 6). The assumptions of multiple linear regression were examined prior to analysis. Residuals were approximately normally distributed. Variance inflation factors (VIFs) were below 5, indicating no problematic multicollinearity. Inspection of residual plots suggested homoscedasticity, and the Durbin–Watson statistic indicated independence of residuals.

The model showed a large effect size (f^2^ Cohen = 0.74), indicating a strong joint explanatory contribution of the predictors to observed mental health risk. Overall, the 95% confidence intervals (95% CIs) indicated statistically significant effects for avoidant (IVIA), secure (Checklist), and sex, as their respective intervals did not include zero, whereas the interval for secure (IVIA) included zero, indicating greater uncertainty regarding its effect.

Avoidant attachment (IVIA) showed a significant positive regression coefficient (β = 0.44, *p* = 0.023), indicating that higher avoidant attachment scores were associated with higher scores of the observational mental health risk indicator within the exploratory model.

Secure attachment-related behaviors observed by professionals showed a significant negative regression coefficient (β = −0.55, *p* = 0.001), indicating that higher levels of observed secure relational behavior were associated with lower scores of the observational mental health risk indicator after adjustment for the other predictors. This direction was consistent with the negative zero-order association between secure checklist scores and the observational mental health risk indicator (ρ = −0.39).

Sex was also a significant predictor (β = 0.36, *p* = 0.027), with female participants showing higher levels of observational mental health risk indicators.

Secure attachment (IVIA) showed a non-significant negative association (β = −0.30, *p* = 0.097).

As an additional sensitivity analysis concerning the age range of the Observation Checklist, the regression was repeated after excluding the nine participants aged 17–18 (n = 24). The reduced-sample model remained statistically significant (R^2^ = 0.438, *p* = 0.022), and the direction of all coefficients was unchanged. Secure checklist remained negatively associated with the observational mental health risk indicator (B = −0.069, *p* = 0.019), and sex remained significant (B = 1.40, *p* = 0.010), whereas avoidant IVIA (B = 0.84, *p* = 0.128) and secure IVIA (B = −0.49, *p* = 0.296) were no longer statistically significant. These results suggest that the overall pattern was broadly retained, although coefficient precision decreased in the smaller sample.

### 3.6. Qualitative Content Analysis of Professional Narratives

The narrative material analyzed in this section was derived from the descriptive evidence fields accompanying the Observation Checklist and was therefore distinct from the separate open-ended question concerning effective support strategies analyzed in Section 3.7. Of the 33 participant records, 17 contained at least one usable narrative entry across these evidence fields.

The inductive qualitative content analysis identified five main categories: (1) behavioral dysregulation and impulsivity, (2) educational engagement and functional adaptation, (3) peer relationships and social vulnerability, (4) attachment needs and support-seeking patterns, and (5) emotional distress and affective dysregulation.

The first category, behavioral dysregulation and impulsivity, included descriptions of low frustration tolerance, oppositional responses to rules, immediate reward seeking, and marked behavioral reactivity. Several adolescents were described as acting impulsively, particularly in situations involving limits, disappointment, or emotional activation, as illustrated by the narrative about P19: “A very impulsive young person, with a constant tendency to satisfy their immediate desires, almost never thinking about the consequences of their actions (small improvements are noted throughout the stay). Quite reactive, especially verbally, particularly when something doesn’t go their way; resorts to threats and insults. Very intensive use of technology and time spent on online games.”

The second category, educational engagement and functional adaptation, reflected a heterogeneous pattern. While some adolescents were described as motivated, engaged, and future-oriented (“She is a young woman with interest, motivation, and high achievement. She has no grade retentions, maintains a good average, and has future goals in higher education. She is well integrated into her class, has friends, and meets the objectives, both individually and as a group.”, P14), others showed poor concentration, low motivation, refusal of academic tasks, and inconsistent participation (“[The young woman] lacks the initiative to do the work. […] has great difficulty expressing herself. […] lacks initiative/interaction with other people.” P15). Educational functioning appeared highly dependent on emotional state, adult support, and contextual structure.

The third category, peer relationships and social vulnerability, captured the adolescents’ ambivalent social functioning. Professionals described both the need to belong and significant interpersonal fragility, including social withdrawal, bullying vulnerability, emotional dependency on peers, and difficulty managing conflict in a balanced way. The narrative about P11 illustrates this category: “Very needy and attached to certain classmates with whom she identifies, she always wants to be with them and have their support and attention, and she dedicates a lot of attention to them. Sometimes she is pushed away for being too intrusive in the support she wants to give. She always tries to join her friends, and when she feels left out, she withdraws and doesn’t complain to the adults, but she shows sadness and dissatisfaction for not being reciprocated with the same intensity of her friendship.”

The fourth category, attachment needs and support-seeking patterns, reflected a strong need for adult reassurance, affection, and emotional containment (“She frequently asks adults for affection; she seeks their attention to be seen; she often complains of “aches” or says she got hurt.” P3). However, support-seeking was often indirect, inconsistent, or selective, suggesting that adolescents frequently struggled to communicate distress openly and relied on relational proximity as a form of regulation.

Finally, the fifth category, emotional distress and affective dysregulation, highlighted anxiety, sadness, emotional lability, concealment of feelings, and behavioral manifestations of distress. Many adolescents appeared to have difficulty identifying and verbalizing emotional experiences, instead expressing distress through withdrawal, agitation, somatic complaints, or disruptive behavior, as illustrated by the narrative of P12: “She tries to hide it and not show how she feels. She says she’s good at pretending everything is okay. She denies her discomfort and suffering. She says she doesn’t trust anyone to truly confide in”.

Overall, the narratives suggest that adolescents in residential care present complex emotional and relational needs, with marked difficulties in self-regulation but also a clear capacity to benefit from consistent, emotionally attuned, and structured caregiving relationships.

### 3.7. Descriptive Summary of Professionals’ Perceptions of Effective Support Strategies

The material analyzed in this section derived from a separate open-ended question asking professionals what they considered to work effectively with each adolescent. Because individual responses could contribute to more than one category, category frequencies were not mutually exclusive and therefore do not sum to the total number of respondents. Given the limited amount of narrative material available for this question, the findings are presented as an exploratory descriptive summary derived from the inductive qualitative content analysis rather than as an in-depth qualitative analysis. Of the 33 records/cases, 8 contained a usable written response and 25 were blank (75.8%). The available responses were organized into five descriptive categories reflecting professionals’ perceptions of effective support strategies.

1. Supportive and Trusting Relationships (n = 6)

Several responses emphasized the importance of building a stable, trusting relationship between the adolescent and the adult caregiver. Professionals reported that improvements often resulted from the gradual development of relational trust, characterized by consistent presence, concern, and persistence from adults. Strategies within this category included active listening, emotional availability, and providing a safe relational space for the adolescent to express feelings. Some participants highlighted the importance of allowing the young person time and space to open up gradually.

2. Emotional Validation and Empathic Communication (n = 5)

Another frequently mentioned category involved acknowledging and validating the adolescent’s emotions while maintaining balanced emotional responses. Respondents reported that humor, calm communication, and non-judgmental listening were effective strategies. This approach allows adolescents to feel understood without reinforcing emotional escalation, facilitating more constructive dialogue and behavioral regulation.

3. Clear Structure, Boundaries, and Guidance (n = 5)

Many responses indicated that adolescents benefit from clear rules, consistent boundaries, and structured guidance. Professionals highlighted the importance of defining clear expectations and roles, communicating instructions clearly and respectfully, and maintaining consistent limits while avoiding judgment. Such structure appears to help adolescents regulate their behavior and understand expectations within the residential environment.

4. Emotional Support and Attachment-Based Care (n = 5)

Several descriptions referred to the importance of emotional closeness and supportive caregiving, including attention, affection, and reassurance. Some responses indicated that physical comfort (e.g., hugs when appropriate within the relationship) and emotional validation can help adolescents feel safer and more regulated. This theme reflects principles consistent with attachment-informed caregiving approaches, emphasizing emotional security as a foundation for behavioral change.

5. Structured Socioemotional Skill Development (n = 2)

A distinct category concerned structured interventions aimed at developing adolescents’ socioemotional competencies, including emotional regulation, social understanding, and interpersonal skills. Professionals referred to programs and educational activities designed to help adolescents recognize and regulate emotions, develop social skills, and understand how their behavior is perceived by others. Unlike the preceding categories, which primarily reflect relational practices embedded in everyday caregiver–adolescent interactions, this category represents a more structured form of developmental and educational support. Although such interventions may complement attachment-informed caregiving by strengthening capacities relevant to relational and emotional functioning, they were not considered attachment-informed relational practices in themselves.

Overall, professionals identified two complementary forms of support: relational caregiving practices embedded in everyday interactions, including trust-building, emotional validation, consistent boundaries, and attachment-sensitive support; and structured socioemotional interventions aimed at developing emotional regulation and interpersonal competencies. The latter should be understood as complementary developmental or educational support rather than as an attachment-informed relational practice in itself.

These findings highlight the importance of relationship-based and trauma-informed practices in residential care settings.

## 4. Discussion

The present study examined attachment-related behaviors and relational dynamics among adolescents living in residential care institutions, with a particular focus on the perceptions of reference adults and their implications for resilience and psychological well-being. The findings highlight the central role of attachment processes in shaping adolescents’ adjustment in residential care and reinforce the importance of emotionally attuned relationships as protective factors in contexts of relational adversity.

Consistent with attachment theory (Bowlby, 1988; Cassidy & Shaver, 2016), the results suggest that attachment representations influence how adolescents perceive, interpret, and respond to relational experiences, during placement in protective care. The predominance of ambivalent and insecure attachment profiles observed in the sample reflects the relational instability and caregiving disruptions commonly experienced by adolescents who experienced relational trauma, the main reason to the implementation of protective measures. These findings are consistent with previous studies reporting elevated levels of attachment insecurity among children and adolescents living in residential care settings (Hillman et al., 2020; Martínez-Usarralde et al., 2025; Schuengel et al., 2014). Nevertheless, approximately one-third of participants demonstrated predominantly secure attachment representations, indicating the presence of adaptive relational expectations despite histories of adversity and residential placement, and may also reflect the potentially beneficial influence of residential care experiences.

One of the most relevant findings concerns the role of avoidant attachment as a predictor of mental health vulnerability. Adolescents presenting avoidant attachment patterns appeared more likely to experience psychological difficulties, supporting previous evidence that avoidant strategies often involve emotional suppression, distrust of others, and reduced help-seeking behaviors (Lyons-Ruth & Jacobvitz, 2016). In residential care contexts, these adolescents may be particularly vulnerable because their distress is frequently less visible to caregivers and professionals. Consequently, their needs may remain unnoticed, limiting opportunities for supportive intervention and emotional co-regulation. Considering the high relevance of ambivalent and avoidant behaviors, these findings reinforce that non-secure attachments are strongly associated with psychopathology (Bosmans & Borelli, 2022).

The negative association between professionally observed secure attachment-related behaviors and the observational mental health risk indicator is consistent with the expected protective role of relational security. Adolescents displaying more secure relational behaviors may show greater capacity to seek support, engage with caregivers, and use interpersonal relationships as a source of emotional regulation. Nevertheless, because both the observational attachment dimensions and the mental health risk indicator were derived from the same professional-rated checklist, this association should be interpreted cautiously and may partly reflect shared-rater or common-method variance.

Adolescents who actively seek reassurance, proximity, or emotional engagement may elicit more frequent responses from caregivers and educators, thereby benefiting from increased opportunities for relational regulation. This interpretation highlights an important distinction between attachment insecurity and relational visibility. In practice, adolescents whose distress is openly expressed may receive greater emotional containment than those whose difficulties remain concealed behind avoidant or withdrawn behaviors.

The findings support the notion that attachment functioning in residential care is heterogeneous and dynamic, with adolescents often exhibiting a combination of secure and insecure relational behaviors depending on situational and relational contexts. However, the simultaneous expression of both patterns is also frequent and may be associated with a greater detrimental impact on the several domains of functioning. Disorganized attachment representations reflect the absence of a coherent strategy for maintaining or restoring a sense of security (Schuengel et al., 1999; van IJzendoorn et al., 2020).

The results indicate that most adolescents were experiencing their first placement in residential care and had an identified reference figure, typically an educator or technical staff member, suggesting the importance of stable relational support within residential care environments. These findings also reinforce the significance of reference adults as potential sources of emotional security and resilience (Babo et al., 2024).

Both quantitative and qualitative data suggest that emotionally available, consistent, and responsive professionals play a crucial role in supporting adolescents’ adjustment. The narratives provided by educators repeatedly emphasized the importance of trust, emotional validation, stable relationships, and sensitive communication. These elements closely reflect the characteristics of attachment-informed caregiving described in the literature (Golding et al., 2016). Rather than functioning solely as supervisors or behavior managers, professionals appear to act as relational regulators who help adolescents develop emotional safety and adaptive coping strategies.

A particularly important contribution of this study concerns the examination of professionals’ perceptions of attachment-related behaviors using an observational tool based on adolescents’ daily behaviors. Understanding how educators interpret adolescents’ behaviors is highly relevant because these perceptions influence daily interactions and intervention strategies. The qualitative findings suggest that professionals who understand behaviors through a relational lens are more likely to respond with empathy, emotional attunement, and consistency, whereas purely behavioral interpretations may risk overlooking underlying attachment needs. These findings support the growing emphasis on attachment-informed and trauma-informed approaches in residential care and reinforce the need for specialized training focused on recognizing attachment signals and relational vulnerabilities.

Another noteworthy finding concerns the weak correlations between adolescents’ self-reported attachment representations and observational measures completed by professionals. This discrepancy may reflect the complex nature of attachment functioning among trauma-exposed populations. Internal working models, emotional experiences, and observable behaviors do not always align in a straightforward manner. However, the significative differences found between secure and non-secure groups concerning observational attachment indicators reveal a convergence aligned with the theoretical principles underneath the measures applied, as in adolescents self-reported as secure professionals observed more secure behaviors, and the reverse was found with the non-secure adolescents. The convergence between these two independent measures highlights that the clinical use of the IVIA and the Observation Checklist may be a useful strategy in order to gain a deeper understanding of relational patterns in residential care.

Adolescents may simultaneously experience needs for closeness and protection while displaying avoidant, defensive, or contradictory behaviors. The variability observed across all attachment-related behavioral domains reinforces the presence of heterogeneous and dynamic relational profiles, suggesting that adolescents in residential care do not conform to uniform attachment patterns but rather exhibit context-dependent behavioral expressions. The use of assessment tools that consider different life settings and dimensions such as the Observation Checklist show that internal working models are sensitive to different settings and relationships and that observed heterogeneity requires a systemic and non-linear approach to uncover regularities, namely the preferred protective strategies during psychological development (Crittenden et al., 2021).

Furthermore, the positive associations observed among the three IVIA dimensions may reflect the coexistence of multiple and partially contradictory internal working models, a phenomenon frequently described among adolescents exposed to chronic relational trauma and caregiving instability. Rather than representing mutually exclusive categories, attachment representations in high-risk populations may coexist in complex and dynamic ways, reflecting the integration of both secure and insecure relational experiences over time. This interpretation is consistent with contemporary attachment perspectives emphasizing that internal working models may remain fragmented, context-dependent, and subject to reorganization following experiences of adversity and subsequent corrective relational experiences (Lyons-Ruth & Jacobvitz, 2016; Van der Kolk, 2014). Such patterns are frequently reported among individuals exposed to chronic relational trauma and may reflect fragmented or mixed internal working models (Cook et al., 2005; Van der Kolk, 2014). These results also highlight the value of combining self-report measures with observational approaches when assessing attachment processes in residential care settings.

The qualitative findings further contribute to understanding the relational complexity of adolescents living in residential care. Professionals described difficulties in emotional regulation, behavioral control, peer relationships, and trust. However, they also identified important strengths, including the capacity to form meaningful relationships, respond positively to emotional support, and benefit from stable caregiving environments. These findings support resilience research emphasizing that positive developmental outcomes can emerge despite significant adversity when children and adolescents have access to supportive and emotionally responsive relationships (Masten, 2014). Taken together, the findings suggest that resilience in residential care may emerge through repeated experiences of emotional safety, trust, and support. The presence of sensitive and attuned reference adults may constitute a protective mechanism through which adolescents can gradually reorganize insecure relational expectations and develop greater psychological well-being (Babo et al., 2024). In this sense, this study reinforces the view that attachment-informed residential care should be understood not only as a protective service but also as a relationally therapeutic environment capable of fostering resilience, emotional regulation, and positive developmental trajectories among young people exposed to relational adversity and implementing recommended best practices (Mishra, 2024).

Importantly, professionals also identified structured socioemotional skill development as beneficial. This category was conceptually distinct from the relational caregiving practices identified in the other categories. Rather than constituting an attachment-informed relational practice, a structured socioemotional intervention may function as a complementary form of developmental support, particularly by strengthening emotional regulation, social understanding, and interpersonal competencies. These findings suggest that effective residential care may benefit from combining relationally sensitive caregiving with structured opportunities for socioemotional skill development.

### 4.1. Limitations

Several limitations should be considered when interpreting these findings.

First, the small sample size (N = 33) substantially limits statistical power, precision, and generalizability. The sensitivity analysis indicated that the regression model was adequately powered only to detect relatively large overall effects; consequently, the regression coefficients may be unstable and sensitive to individual observations. The regression findings should therefore be regarded as preliminary and exploratory.

Second, the cross-sectional design precludes causal interpretations. Third, the Observation Checklist is a reflective and monitoring instrument rather than a diagnostic mental health measure; accordingly, the mental health risk score used in this study should be interpreted only as an exploratory observational indicator of psychological vulnerability. Finally, the qualitative material was limited and heterogeneous, with a substantial proportion of blank responses for the question concerning effective strategies. Although coding was conducted manually by two researchers and differences were resolved through consensus, the qualitative findings should be regarded as exploratory descriptive findings rather than as an in-depth qualitative account.

Third, the reliance on professional reports for observational data may introduce subjective bias. Future research should employ larger samples, multi-informant designs, and longitudinal approaches to further clarify these relationships. Longitudinal studies are particularly needed to examine how attachment representations evolve over time and whether changes in caregiving relationships contribute to improvements in resilience and psychological well-being.

Fourth, participants were recruited from five residential care institutions and were assessed by 20 professionals. Because some adolescents may have shared the same institutional context and/or evaluator, complete independence of observations cannot be assumed. The small number of institutions and the limited overall sample precluded reliable estimation of multilevel or cluster-adjusted models. Consequently, the regression estimates may be sensitive to within-institution or within-evaluator dependence and should be regarded as preliminary. The leave-one-institution-out sensitivity analyses further showed that although the direction of the regression coefficients remained consistent across all reduced-sample models, their magnitude and statistical significance varied, indicating limited precision and some sensitivity to sample composition. Future studies with larger samples and a greater number of institutions and evaluators should explicitly model this nested data structure.

Fifth, the instrument was originally developed for young people aged 11–16 years, whereas nine participants in the present sample were aged 17–18. Although sensitivity analysis indicated that exclusion of these participants did not alter the direction of the regression coefficients and the overall model remained significant, the validity of the checklist for this older age range has not been established. Findings involving these participants should therefore be interpreted cautiously, and future studies should examine the applicability and measurement properties of the checklist in older adolescents.

Finally, a further limitation concerns shared-rater and common-method effects. The observed attachment-related dimensions and the observational mental health risk indicator were derived from the same checklist and rated by the same professional for each adolescent. Associations between these professional-rated variables may therefore partly reflect shared method variance or rater-specific perceptions rather than associations between fully independent measures. Future studies should incorporate independent raters and/or additional sources of mental health assessment to reduce common-method effects.

### 4.2. Implications for Practice

The findings of this study have important implications for residential care practice, particularly regarding the implementation of attachment-informed and trauma-informed approaches. The results suggest that adolescents’ attachment-related behaviors cannot be adequately understood solely through behavioral observation; rather, they must be interpreted within the context of their relational histories and internal working models. Consequently, residential care professionals require specialized training that enables them to recognize attachment signals, understand the relational meaning underlying behavior, and respond in ways that promote emotional security and resilience.

Particular attention should be given to adolescents displaying avoidant attachment patterns. The findings indicate that these young people may be at greater risk of experiencing psychological difficulties while simultaneously being less likely to seek help or express distress openly. As a result, professionals should be trained to identify less visible forms of vulnerability and to adopt proactive relational strategies that encourage trust, emotional expression, and help-seeking behaviors.

This study also highlights the importance of reference adults within residential care settings. Stable, emotionally available, and responsive relationships appear to function as key protective mechanisms that support resilience and socioemotional adjustment. Residential care organizations should therefore prioritize relational continuity, minimize unnecessary changes in caregiving staff, and promote the establishment of meaningful long-term relationships between adolescents and designated reference adults. Such practices may strengthen adolescents’ sense of safety, belonging, and emotional security.

Furthermore, the qualitative findings emphasize the value of emotional validation, empathic communication, relational consistency, and clear but supportive boundaries. These elements are consistent with attachment-informed caregiving frameworks and should be integrated into daily residential care practices. Interventions should move beyond behavior management approaches and incorporate strategies that promote co-regulation, reflective functioning, emotional literacy, and the development of trusting relationships.

The discrepancy observed between self-reported attachment representations and professionals’ observations further suggests the need for comprehensive assessment procedures that combine multiple sources of information. Residential care services should consider the use of validated attachment assessment tools alongside professional observations to obtain a more accurate understanding of adolescents’ emotional and relational functioning.

Finally, the qualitative findings reinforce the view that resilience is fundamentally relational. Residential care environments that foster emotional safety, predictable relationships, and opportunities for positive relational experiences are more likely to promote adaptive developmental trajectories and psychological well-being. Investment in attachment-informed training and assessment tools, reflective supervision, and relationship-based practice may therefore represent one of the most effective strategies for enhancing outcomes among adolescents living in residential care.

## 5. Conclusions

This study contributes to the growing body of research on attachment processes in residential care by highlighting the central role of relational dynamics and reference adults in supporting adolescents exposed to adversity. Based on a multi-method and multi-informant approach, it contributes to filling the gap between attachment theory and professional practices in residential care.

The findings reveal a predominance of insecure attachment patterns, alongside considerable heterogeneity in both self-reported attachment representations and observed attachment-related behaviors, and suggest that resilience in residential care may develop through stable, supportive, and emotionally attuned interactions with caregivers who are capable of providing emotional security, validation, consistency, and opportunities for co-regulation.

It highlights the value of attachment-informed and trauma-informed approaches in residential care, emphasizing the need for professionals to understand the relational meanings underlying adolescents’ behaviors (rather than focusing mostly on behavior management) and to engage in relational (rather than behavioral management) intervention strategies. The gap between attachment theory and attachment-based practices can be successfully addressed through the use of this multi-informant and multi-method approach.

Although exploratory in nature and limited by its sample size, the findings provide important insights into the mechanisms through which residential care can promote psychological well-being, emotional regulation, and resilience. Future research should employ larger and longitudinal samples to further examine how attachment processes evolve over time and how relationship-based interventions may contribute to positive developmental trajectories among adolescents living in residential care.

## Figures and Tables

**Table 1:** Sociodemographic characteristics of adolescent sample.

Variable	Category/Statistic	n (%) or *M*
Age (years)	Mean	15.0
	Range	12–18
Sex	Female	18 (54.5%)
	Male	15 (45.5%)
School grade	5th grade	1 (3.03%)
	6th grade	2 (6.1%)
	7th grade	2 (6.1%)
	8th grade	7 (21.2%)
	9th grade	2 (6.1%)
	10th grade	5 (15.2%)
	11th grade	3 (9.1%)
	12th grade	1 (3.0%)
	missing	9 (27.3%)
Special educational needs	No identified SEN	29 (87.9%)
	ADHD	2 (6.1%)
	Intellectual functioning difficulties	1 (3.0%)
	Specialized educational support	1 (3.0%)
Length of stay in residential care	Mean	24 months
	Range	1 month–8 years
Previous residential placement	No	25 (75.8%)

**Table 2:** Relational and social characteristics of adolescents in residential care.

Category	Response	n	%
Previous residential placement	No	25	75.8
	Yes	8	24.2
Number of previous placements	One	7	87.5
	Two	1	12.5
Reference figure in residential care	Yes	30	90.9
	No	3	9.1
Feels listened to by professionals	Always	20	60.6
	Sometimes	12	36.4
	Rarely	1	3.0
Trust in professionals	Yes	18	54.5
	Depends on the situation	14	42.4
	No	1	3.0
Relationships with other adolescents	Very good	6	18.2
	Good	17	51.5
	Regular	10	30.3
Contact with family	Daily	20	60.6
	Weekly	9	27.3
	Occasionally	2	6.1
	No contact	2	6.1
Seeks support when feeling sad	Yes	25	75.8
	No	8	24.2
External support available	Yes	30	90.9
	No	3	9.1

**Table 3:** Attachment representations assessed using the Inventory of Attachment in Childhood and Adolescence (IVIA).

Attachment Dimension	n (%)	M (SD)	Min.	Max.
Secure IVIA	11 (33.3)	3.07 (0.73)	1.75	4.63
Ambivalent IVIA	15 (45.5)	3.19 (0.61)	2.25	4.13
Avoidant IVIA	5 (15.2)	2.97 (0.63)	1.88	4.50
No predominant classification *	2 (6.1)			

Note: * 2 adolescents show the same punctuation in all dimensions.

**Table 4:** Descriptive statistics for the observational checklist domains.

Attachment Dimension	M (SD)	Min.	Max.
Secure Checklist	19.82 (8.54)	5	37
Ambivalent Checklist	11.91 (7.10)	3	26
Avoidant Checklist	11.58 (6.47)	2	24

**Table 5:** Attachment-related behaviors and socioemotional functioning: Observation Checklist.

Variables	1	2	3	4	5	6	7	8
1. Observational Mental Health Risk Indicators	—	0.41	−0.39	0.36	0.05	−0.28	−0.10	0.34
2. Ambivalent (Checklist)		—	−0.71 **	−0.11	0.04	0.28	0.20	0.18
3. Secure (Checklist)			—	−0.62 **	−0.06	−0.16	−0.08	−0.21
4. Avoidant (Checklist)				—	0.10	−0.10	−0.11	0.09
5. Time in Care					—	−0.22	0.03	−0.21
6. Secure (IVIA)						—	0.58 **	0.52 **
7. Avoidant (IVIA)							—	0.63 **
8. Ambivalent (IVIA)								—

Note: ** Correlation significant for *p* < 0.001.

**Table 6:** Multiple linear regression predicting observational mental health risk indicators.

Predictor	B	SE	β	*t*	*p*	*CI*_95%_
Constant	2.27	1.18	—	1.92	0.065	
Avoidant (IVIA)	1.10	0.46	0.44	2.41	0.023	0.16; 2.04
Secure (IVIA)	−0.62	0.36	−0.30	−1.72	0.097	−1.36; 0.12
Secure (Checklist)	−0.09	0.02	−0.55	−3.81	0.001	−0.14; −0.04
Sex	1.05	0.45	0.36	2.33	0.027	0.13; 1.97

## Data Availability

Data are unavailable due to privacy and ethical restrictions.
